# Patterns of Neuropsychiatric Manifestations of Ovarian Teratomas: A Systematic Review of Case Reports

**DOI:** 10.7759/cureus.67190

**Published:** 2024-08-19

**Authors:** Gaelle Umutoni Mihigo, Luigjina Uljic, Jasrina Kaushal, Shannia Amoah, Kudrat Jha, Ayodeji Jolayemi

**Affiliations:** 1 Medicine, American University of Antigua, St. John's, ATG; 2 Internal Medicine, Medical University of the Americas, Charlestown, KNA; 3 Psychiatry, Interfaith Medical Center, Brooklyn, USA

**Keywords:** neuropsychiatric, limbic encephalitis, psychosis, case report, psych, psychiatric, psychiatric symptoms, teratoma, ovarian teratoma

## Abstract

Ovarian teratomas are germ cell tumors composed of multiple cell types. Ovarian teratomas may express antigens found in the nervous system or neuroendocrine proteins. These neural antigens and neuroendocrine proteins may lead to an autoimmune response with associated encephalitis. There are a growing number of case reports of autoimmune encephalitis in patients with ovarian teratomas. However, the patterns of neuropsychiatric manifestations of ovarian teratomas associated with encephalitis have not been established. The aim of this article is to conduct a systematic review to determine the patterns of neuropsychiatric manifestations of ovarian teratoma-associated encephalitis, focusing on their frequency and clinical course. Thirty-three case reports were collected and analyzed for a systematic review. The studies were full-text, peer-reviewed journal publications from April 2014 to April 2024. Fifty-eight patients were included in our study. The age group of 22-35 years old was the most reported, with 25 (43.1%) patients. The most commonly reported symptoms were memory impairment in 29 (50%) patients, hallucinations in 25 (43.1%) patients, and aggressive behavior in 23 (39.7%) patients. Neuropsychiatric symptoms had a prodromal phase of flu-like symptoms in 31 (53.4%) patients. The neuropsychiatric symptoms preceded the diagnosis of ovarian teratoma in 57 (98.3%) patients. In 53 (91.4%) patients, patients did not respond to psychiatric medications. Autoimmune antibodies to neural antigens were found in 45 (77.6%) patients, with 25 (43.1%) patients having neural tissue present in the teratoma. Treatment of the underlying teratoma and encephalitis led to full recovery in 37 (63.8%) patients. However, long-term outcomes such as relapse and mortality were discussed in only 11 (19.0%) patients. Findings suggest that neuropsychiatric symptoms correlate with teratoma-associated encephalitis and often precede tumor detection. The treatment of the teratoma led to full recovery of the neuropsychiatric manifestations; however, the long-term outcomes of the patients need to be further studied. Future research is needed on the prognosis of patients with neuropsychiatric manifestations of ovarian teratoma.

## Introduction and background

Ovarian teratomas are the most common germ cell tumors of the ovary. They are found in up to 95% of all ovarian germ cell tumors [1]. A majority of ovarian teratomas are mature and benign tumors [2]. Mature ovarian teratomas are usually cystic, such as mature cystic teratomas or dermoid cysts. Solid tumors, however, can occur. These benign tumors usually have a well-ordered, “organoid” appearance of the differentiated tissues within the teratoma and the absence of cytologic atypia [2]. These differentiated tissues include neural tissue. Immature teratomas are rare and consist of immature-appearing tissues, principally neuroepithelium [1]. Ovarian teratomas have been associated with a number of complications, including but not limited to autoimmune hemolytic anemia in 1% of cases, hyperthyroidism in 0.5% of cases, carcinoid syndrome in 0.7% of cases, and autoimmune encephalitis in 1% of cases [1,3]. These complications are more commonly seen in mature and cystic teratomas and less commonly in immature and solid teratomas [2,3]. Autoimmune encephalitis is the focus of this review.

Encephalitis due to ovarian teratomas was first described by Dalmau et al. in their case series of women presenting with severe nonviral encephalitis [4]. Subsequent to this, there have been an increasing number of case reports on ovarian teratoma-associated encephalitis [5]. Although theories on the pathogenesis of encephalitis in teratomas are still in the early stages, positive antibodies to neural elements in the serum and cerebrospinal fluid appear to be a common finding [3]. The initial clinical presentations of the cases are usually psychiatric symptoms without other physical findings [3]. Due to the rare nature of this disorder, many cases are often misdiagnosed as psychiatric disorders [5]. A review of the various case reports for patterns of neuropsychiatric manifestations will be of importance in aiding patient diagnosis [5]. We therefore present a systematic review of case reports and case series of ovarian teratoma presenting with neuropsychiatric manifestations. We discuss the nature and frequency of various neuropsychiatric symptoms, their course, and patterns of results from investigational studies such as laboratory and imaging findings. Clinical management and outcomes are also discussed.

In performing this review, we take into consideration the association between ovarian teratomas and antibodies to neural tissues in understanding the neuropsychiatric manifestations that present. We also take into consideration the regions of the brain involved in autoimmune reactions to neural tissues in understanding the neuropsychiatric manifestations. In terms of antibodies to neural tissues, studies have shown that some patients with teratomas have antibodies to N-methyl D-aspartate receptors (NMDAR) [4]. Hence, an immune-mediated mechanism of dysfunction of NMDAR may be important in the pathogenesis of encephalitis associated with teratomas [4]. The autoimmune mechanism of interest involves antibodies to heteromers of NR2C and NR2D subunits of NMDAR [6]. Although the mechanisms triggering the development of antibodies to NMDAR are unclear, studies have shown the ectopic expression of NR2 subunits by nervous tissue contained in the teratomas [3]. These antibodies to NMDAR then breach the blood-brain barriers, causing autoimmune reactions in various regions of the brain [4].

Brain imaging studies such as MRI have shown that the regions of the brain involved include the hippocampus, amygdala, medial temporal lobes, frontal lobes, the cerebellum, the brainstem, and the basal ganglia [3]. These brain regions are usually involved because the blood-brain barrier is more vulnerable in these regions [4]. According to theories of the pathogenesis of encephalitis, the autoimmune reactions in these regions result in clinical presentations consistent with their neurobehavioral functions [6]. For instance, the hippocampus plays a role in memory formation, and learning with NMDA receptors is important in long-term potentiation [4]. Antibodies to NMDA receptors could therefore lead to cognitive impairments in patients. Similarly, the NMDA receptor mediates glutamate signaling in the cortex [7]. Autoimmune antibodies could result in NMDAR hypofunction, leading to paranoia, hallucinations, and dyskinesias [4]. However, correlating the neuropsychiatric manifestations of teratoma-associated encephalitis with the neurobehavioral functions of the brain regions likely involved in autoimmune reactions has yet to be done. This review, which also looks at the patterns of neuropsychiatric symptoms, could provide a foundation to correlate the clinical symptoms of teratoma-associated encephalitis with the involved brain regions and their neurobehavioral functions.

Understanding the patterns of neuropsychiatric symptoms in patients may also play a role in the early diagnosis of teratomas, as these symptoms often precede the diagnosis of teratomas [3]. Psychiatrists also do not often acknowledge ovarian teratomas in the differential diagnosis of female psychiatric patients, especially in cases refractory to psychiatric medications [5]. The diagnosis of teratoma-associated psychiatric disorders is on average delayed by 16 months due to patients being misdiagnosed as having schizophrenia that is poorly responsive to medications [4,5]. The results of this review may lead to improvements in the diagnosis of ovarian teratoma-associated encephalitis, both for early tumor diagnosis and to prevent misdiagnosis of the disorder.

## Review

Methods

We conducted a literature review from May 2024 to July 2024 on case reports of women aged two to 90 years old presenting with neuropsychiatric manifestations of ovarian teratoma. Reviewers from different affiliations met in person for collaboration when they shared a common hospital site of clinical rotation. In addition, collaboration continued online after clinical rotations through the use of shared online documents and conferencing applications. The collected data was inputted into a structured Google sheet, with all team members having access to the Google sheet. The Google sheet was securely managed and shared exclusively among the group members to maintain confidentiality and data integrity.

The retrieved information then went through a series of reviews each week by different reviewers for a duration of two months. The Preferred Reporting Items for Systematic reviews and Meta-Analyses (PRISMA) guidelines were followed to identify study populations accurately. Literature reviews were conducted using PubMed/MEDLINE, Google Scholar, Scopus, Ovid, JoVE, EBSCO, ProQuest, DOAJ, and ScienceDirect. The research was conducted using search terms including but not limited to “Ovarian teratoma”, “Teratoma,” “Psychiatric symptoms,” “Psychiatric,” “Psych,” “Case report,” “Psychosis,” “Limbic encephalitis,” and “Neuropsychiatric.” These search terms were used in all possible combinations.

Case reports were included if they were published after April 2014, confirmed cases of ovarian teratoma, and manifested any psychiatric manifestations (i.e., hallucinations, paranoid delusions, anxiety, depression, mania, bizarre behavior, or altered mental status). All case reports were eligible for inclusion if the patient was between two and 90 years old. Cases without imaging or histopathology results suggesting teratoma were excluded from the final analysis. Race and gender were not exclusion criteria and were included in the literature review. The presence of neurological symptoms was considered, but it did not alter the inclusion criteria.

A total of 72 case reports/case series were recorded through database searching. This resulted in a total of 67 case reports remaining after duplicates were removed. After careful screening, 34 case reports were excluded from the review for the following reasons: they were not case reports, lacked sufficient information, had no imaging or histopathological evidence of ovarian teratoma, presented neurological but not psychiatric symptoms, or had inaccessible full-text articles. Thirty-three separate case reports were used, resulting in 58 total patients analyzed for the paper. Of note, the presence of case series in our analysis can account for the higher patient volume than that of case reports. 

Case reports went through an extensive process with six people searching case reports, retrieving relevant information, and paraphrasing key points for analysis in Excel spreadsheets. Any discrepancies were addressed by all authors. There was no direct contact with any of the authors of the literature review articles. Although no statistical analysis was done to analyze the results, two authors calculated the frequencies of the psychiatric symptoms, neuroimaging findings, serological findings, histopathology, and therapeutic interventions.

**Figure 1:**
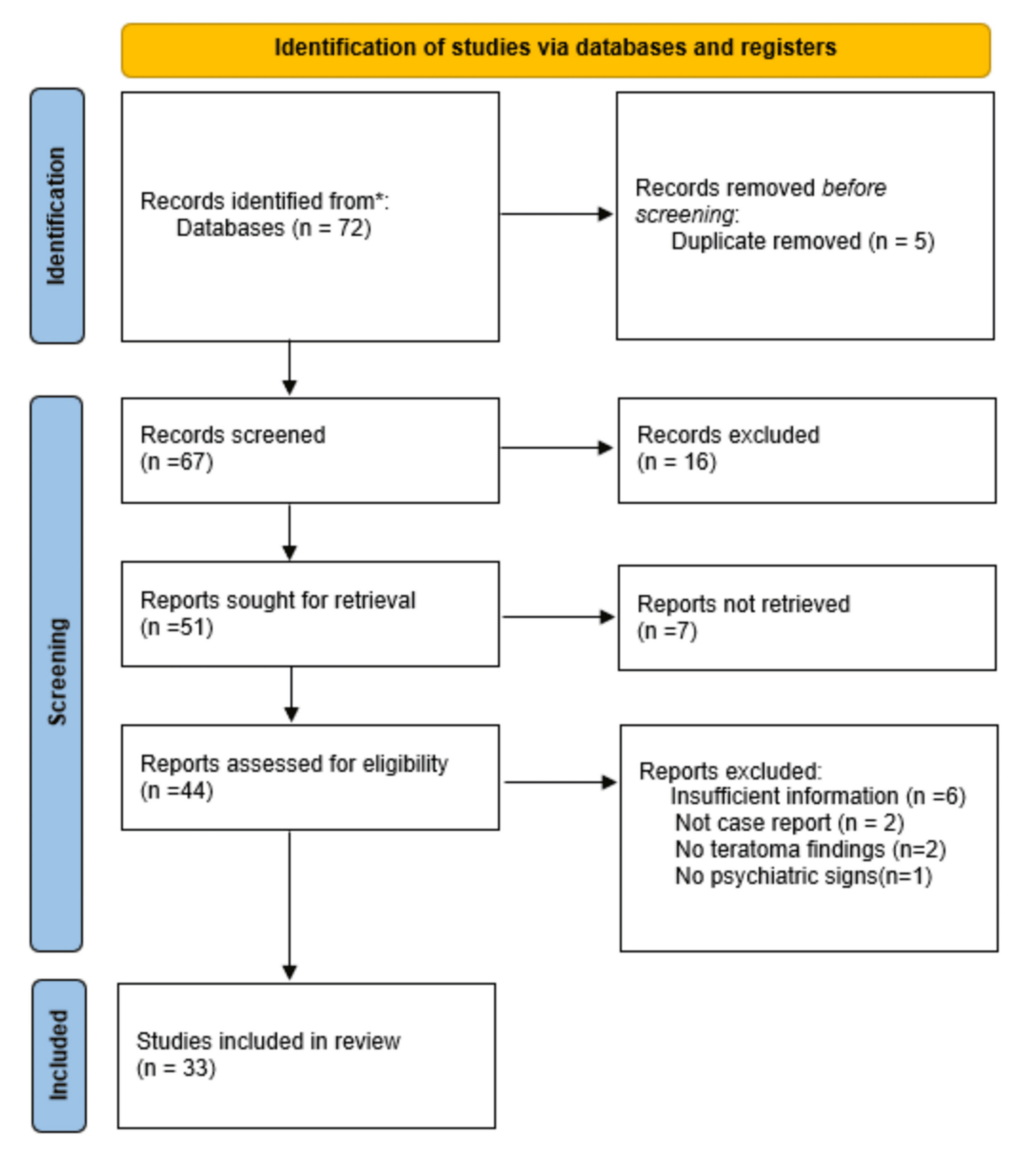
PRISMA flowchart PRISMA, Preferred Reporting Items for Systematic reviews and Meta-Analyses

A PRISMA flowchart of the case report search, as described before, is shown in Figure 1.

Results

Thirty-three studies with a total of 58 patients were included in the final analysis [8-40]. There were potential areas of bias in these individual case reports that were analyzed, for instance, grant support [8-10]. All other papers reported no conflicts of interest. To determine the risk of bias in the case reports, all other case reports were critically appraised using the Joanna Briggs Institute (JBI) critical appraisal checklist for case reports [41]. The results of the appraisal showed that all case reports met reporting standards with no impact of potential conflicts of interest on the outcome of our results.

The demographic characteristics of patients are presented in Table 1. The case reports were all female patients with ages ranging from 12 years to 45 years.

**Table 1:** Demographic characteristics of the neuropsychiatric manifestations of encephalitis associated with ovarian teratoma

	Age							
	2-10	10-13	14-17	18-21	22-35	36-44	45-64	65+
Number	0	1	11	12	25	7	1	0
Percentage	0	1.7	19	20.7	43.1	12.1	1.7	0

When looking at each group of the female population in the early adulthood range, 22-35 were the most reported, with 25 (43.1%) patients. This was more than double the other age ranges. The next age range group that was reported the most was the late adolescent range (18-21). A total of 12 (20.7%) late-adolescent patients were reported. This was followed by the middle adolescent age range (14-17), with 11 (19.0%) reported patients. The early middle age range (36-44) was the next most reported age range, with seven (12.1%) patients. Early adolescence and late middle age were the next reported age groups, each with one (1.7%) patient. The least reported in the case reports were age ranges of two to 10 years and 65 years, with no patients reported.

The number of patients reported for each age range increased from one (1.7%) patient in the age range of 10-13 years to 11 (19.0%) patients in the age range of 14-17 years. This then increased to 12 (20.7%) reported patients in the age range of 18-21 years. The increase peaked at 25 (43.1%) patients in the age range of 22-34 years. After the peak, the number of reported patients decreased to seven (12.1%) patients in the age range of 36-44 years, with a further decrease to one (1.7%) patient in the age range of 45-64 years. The number of reported patients was lowest at the age extremes.

In terms of prodromal symptoms to the neuropsychiatric manifestations, 31 (53.4%) patients reported flu-like symptoms preceding the onset of behavioral disturbances. The flu-like symptoms reported included fever, cough, runny nose, muscle aches, headaches, and weakness. A total of 27 (46.6%) patients did not report any flu-like symptoms.

**Figure 2:**
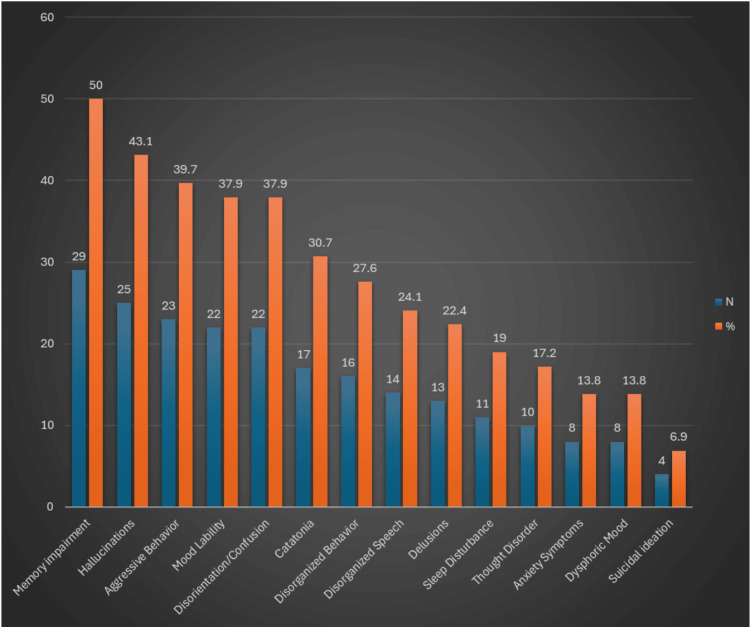
Neuropsychiatric manifestations of ovarian teratoma-associated encephalitis

The frequency of psychiatric manifestations in the patient population is displayed in Table 2 and Figure 2. The most frequent symptom was memory impairment, reported in 29 (50%) patients. The memory impairment was reported to be temporary in nature, lasting the duration of the illness. The second most frequent symptom was hallucinations, reported in 25 (43.1%) patients. The third most common symptom was aggressive behavior, reported in 23 (39.7%) patients. Mood lability was the next most frequently reported symptom reported in 22 (37.9%) patients. A similar number of 22 (37.9%) patients were reported to have disorientation/confusion. The next neuropsychiatric manifestation was catatonia, which was reported in 17 (30.7%) patients. This was followed by disorganized behavior, which was reported in 16 (27.6%) patients. Disorganized speech followed closely behind and was reported in 14 (24.1%) patients. Delusions, including persecutory delusions, were seen in 13 (22.4%) patients. Sleep disturbances were also seen in 11 (19%) patients. Dysphoria was reported in eight (13.8%) patients, while suicidal ideations were reported in four (6.9%) patients. Of note, dysphoria and suicidal ideation preceded the patient being diagnosed with an ovarian teratoma.

**Table 2:** Neuropsychiatric manifestations of ovarian teratoma-associated encephalitis

Clinical presentation	N	%
Memory impairment	29	50
Hallucinations	25	43.1
Aggressive behavior	23	39.7
Mood lability	22	37.9
Disorientation/confusion	22	37.9
Catatonia	17	30.7
Disorganized behavior	16	27.6
Disorganized speech	14	24.1
Delusions	13	22.4
Sleep disturbance	11	19
Thought disorder	10	17.2
Anxiety symptoms	8	13.8
Dysphoric mood	8	13.8
Suicidal ideation	4	6.9

We observed the timing of psychiatric symptoms due to encephalitis to determine if they were noticed before the diagnosis of ovarian teratoma. The results showed that in 57 (98.3%) patients, neuropsychiatric symptoms due to encephalitis were reported before the diagnosis of ovarian teratoma. The average time taken to diagnose ovarian teratoma from the onset of neuropsychiatric symptoms was 26.8 days. We observed that the average time taken to diagnose teratoma varied for the different neuropsychiatric symptoms. The lowest time to diagnosis was seen in seizures, with an average time to teratoma diagnosis of 21.4 days, and in catatonia, with an average of 25.9 days to diagnosis of teratoma. Longer times to diagnosis of teratoma were seen in patients with hallucinations with an average of 29.3 days, memory impairment with an average of 32.8 days, mood lability with an average of 31.1 days, depressed mood with an average of 36.7 days, and anxiety with an average of 41.9 days. Neurological symptoms were reported in 50 (86.2%) patients. The most common neurological symptom reported was seizures in 38 (65.5%) patients. However, despite neurological symptoms being present, only 15 (25.9%) showed abnormalities in the neurological workup, such as imaging and EEG. Among these, three (5.2%) patients had abnormalities in the temporal lobe.

The investigational studies confirming encephalitis and associated ovarian teratomas were also observed. In laboratory studies supporting encephalitis, 45 (77.6%) patients were reported to have antibodies to NMDAR in both their CSF and serum. However, workups for other types of autoimmune antibodies, such as anti-Hu and anti-MA2, did not reveal any positive findings. The imaging findings for ovarian teratoma revealed a cystic lesion in 33 (56.9%) patients, with solid tumors reported in four (6.9%) patients. Pathology studies of the tumor after resection revealed mature tumors in 43 (74.1%) patients, with immature tumors reported in 10 (17.2%) patients. Malignancy was present in only seven (12.1%) patients. Nervous tissue was reported to be present in the teratoma of 25 (43.1%) patients.

In terms of clinical management, the neuropsychiatric symptoms did not respond to psychiatric medications in 53 (91.4%) patients. A total of 57 (98.3%) patients were treated with surgical resection of the teratoma. Patients also received steroid treatment in 49 (84.5%) cases, intravenous immunoglobulins in 35 (60.3%) cases, plasma exchange in 17 (29.3%) cases, and rituximab in 15 (25.9%) cases. Following these treatment interventions, 37 (63.8%) patients were reported to have completely improved and resumed premorbid functioning. The average time taken from the onset of psychiatric symptoms to the removal of teratoma for the 37 (63.8%) showing complete improvement was 42.7 days. Of the 21 (36.2%) patients who did not show complete improvement, the average time taken to remove the teratoma was 44.2 days. Of note, post-recovery long-term outcomes were discussed in only 11 (19.0%) of the patients. Of these cases, four (6.9%) remained collapse-free at 48 months. The average time the teratoma was removed following the onset of psychiatric symptoms in the 4 (6.9%) cases that remained collapse-free was 41.2 days. For the seven (13.1%) cases that had long-term outcomes of relapses, the average time the teratoma was removed following the onset of psychiatric symptoms was 44.1 days.

Discussion

We report the presence of psychiatric manifestations in patients who have encephalitis associated with ovarian teratomas with no prior history of any psychiatric conditions. A timeline of the neuropsychiatric manifestations of many of the patients can be seen from the results. The clinical course in many reported cases began with a flu-like illness, including symptoms such as headaches, coughs, fevers, and muscle aches. This is then followed by the emergence of psychiatric symptoms. The most commonly reported presentations include memory impairment. It is important to note that the hippocampus is one of the regions identified in the pathogenesis of teratoma-associated encephalitis due to the vulnerability of its blood-brain barriers to anti-NMDAR antibodies [2]. Hallucination was the next most common neuropsychiatric manifestation, which is also consistent with NMDAR hypofunction and disrupted glutamate signaling due to anti-NMDAR antibodies [4,7]. The next most common symptom was aggressive behavior. The amygdala, which plays a role in the emergence of aggressive behavior [42], is also one of the brain regions noted for the vulnerability of its blood-brain barriers to anti-NMDAR antibodies [2]. The presence of disorientation was also common and likely indicates the presence of delirium [43]. Catatonia was reported in a number of cases. The cortico-basal ganglia loops, which are thought to play a role in catatonia [44], have also been identified as brain regions targeted by anti-NMDAR antibodies [2]. Affective symptoms are also reported, including mood lability and dysphoria. Some patients also report suicidal ideation. It is important to note that these neuropsychiatric symptoms are often accompanied by neurological symptoms, with seizures and disturbances in consciousness being the most common.

In terms of clinical course, the psychiatric symptoms do not improve with the use of psychiatric medications. Neurology work, including MRI and EEG, does not demonstrate abnormalities in most cases. Imaging of the abdomen and/or pelvis using ultrasound or CT scans revealed ovarian teratomas, with most cases being cystic. Malignancy was rare in these cases. Histopathological examination of the teratomas typically showed mature teratomas, and nervous tissue was reported in many of them. The presence of neural tissue may provide some understanding of the pathogenesis of encephalitis in patients. Current theories of pathogenesis note that ovarian teratomas have some sporadic neuroglial cells, tertiary lymphoid structure, and inflammatory infiltrates in addition to the teratoma tumor cells [45]. It is believed that the process begins with the expression of NMDARs on the surface of ovarian teratoma cells [45,46]. Mature dendritic cells present in the teratoma capture neural antigens of NMDARs produced and present antigenic fragments to T cells, resulting in the induction of T cell activation, differentiation, and proliferation [45]. The activated CD4+ T cells then induce the differentiation of B cells into plasma cells, with the subsequent generation of IgG autoantibodies [47]. The immunocytes and autoantibodies then circulate in the bloodstream and lymphatic systems and cross the blood-brain barrier into the CSF [47].

The role of circulating immunocytes and anti-NMDAR antibodies in pathogenesis seems to be consistent with the findings in our review. Abnormal findings were seen in CSF and serum tests for autoimmune antibodies in most patients. The most common antibodies found in the patients are anti-NMDAR. Of note, studies have shown a correlation between a diagnosis of ovarian teratoma-associated limbic encephalitis and the presence of positive CSF NMDAR antibodies [48]. The demonstration of IgG antibodies against the NR1 subunit of the NMDA receptor in the CSF is more sensitive and specific than serum testing for anti-NMDAR antibodies [45]. To avoid false-negative or false-positive results, both CSF and serum are tested for NMDAR antibodies [45]. Although other antibodies may be present [48], none of the cases in this review reported other autoimmune antibodies. In addition to positive anti-NMDAR test results, the patient’s CSF may have nonspecific abnormalities. These abnormalities include mild pleocytosis and mildly increased protein [47].

It is interesting to note that most patients in the review were treated with surgical resection of the tumor. In addition, surgical resection was combined with corticosteroids, intravenous immunoglobulins (IVIGs), plasma exchange, or rituximab. The current consensus on the management of limbic encephalitis considers corticosteroids as a first-line agent for anti-NMDAR encephalitis [49]. Steroids alone may be insufficient, and they may be used in conjunction with IVIGs and/or plasma exchange [49]. Gong et al. found that IVIGs plus intravenous methylprednisolone had a higher response rate compared to either alone [50]. Suppiej et al. also observed a trend toward better outcomes at the last follow-up when plasma exchange was combined with corticosteroids [51]. It is interesting to note that rituximab, which was also used by some patients in the review, has some studies showing patient improvements. Rituximab is a B-cell-depleting, partially humanized monoclonal antibody directed against CD20 [52]. Its use is consistent with theories of the pathogenesis of encephalitis, as described earlier in this discussion. Nosadini et al. showed that rituximab was associated with reduced relapses in patients with anti-NMDAR encephalitis [53]. A German registry cohort with a majority of patients having anti-NMDAR encephalitis found that fewer relapses occurred after rituximab treatment [54].

Although most patients in the study fully recovered following treatment, the long-term prognosis of the patients may be of concern. In a study by Zhang et al. [55], patients experienced multiple relapses during a median follow-up period of 46 months. Relapses include recurrent seizures, memory deficits, pulmonary metastasis of the tumor, pancreatitis, and multiple organ dysfunction syndromes. Deaths were also reported in the follow-up study [55]. Two predictors of good outcomes included the lower severity of symptoms and the prompt initiation of treatment, including tumor removal [56]. Patients who do not improve with first-line therapy within the first four weeks of starting treatment may have a poorer prognosis [57]. These findings emphasize the importance of early detection of teratomas. The possibility of using neuropsychiatric manifestations, which usually precede the diagnosis of teratoma, may be of interest for future research.

It is important to acknowledge and appraise the risk of bias in this review. The first bias worth discussing is the bias in the individual studies. As acknowledged in the results section, we noted potential areas of bias in the individual case reports, including grant support [8-10]. There is a risk that such a grant may affect the data included in the case report. To assess the risk of bias in individual cases, we used the JBI critical appraisal checklist for case reports [41]. All case reports were scored critically on the checklist. It was determined that the potential for bias did not impact the results of the review.

In addition to the risk of bias due to individual cases, there was the potential for bias in the systematic review process. The first is the risk of not including case reports that should have been included in the systematic review. This can be due to some case reports not being indexed in popular databases due to either publication bias, time-lag bias, language bias, or location bias. We acknowledged this potential for bias by including an exhaustive number of databases in our search, including PubMed/MEDLINE, Google Scholar, Scopus, Ovid, JoVE, EBSCO, ProQuest, DOAJ, and ScienceDirect. There is also the risk of selective outcome reporting bias. The use of the PRISMA guidelines in this review provides some measure of mitigation against this bias.

In terms of future directions, it is important to note that this review includes only 58 patients. This small number is expected, given that encephalitis due to ovarian teratoma is very rare. According to Yaguchi et al., encephalitis due to ovarian teratoma occurs in just 1.17% of teratoma cases [58]. It may be difficult to draw clear conclusions on long-term clinical outcomes given the sample size. It may be possible, however, to conduct future studies looking at the different aspects of ovarian teratoma-associated encephalitis as more cases are reported. For instance, a review with a larger sample size focusing on the neuropsychiatric manifestations of ovarian teratoma and clinical outcomes may provide significant clinical results.

## Conclusions

Ovarian teratomas can be associated with encephalitis, which may present with neuropsychiatric symptoms. These symptoms often precede the diagnosis of the teratoma and may be preceded by prodromal flu-like symptoms. These symptoms may not respond to psychiatric medications but usually resolve with surgical resection in combination with immunotherapy. A positive NMDAR is seen in many of the patients and may be important in the pathogenesis. The diagnosis is also established by imaging and histopathological findings, which may show neural tissue present. Full recovery is observed in many patients following treatment; however, the long-term prognosis remains uncertain. Future studies are needed to establish the long-term prognosis of patients following recovery.
